# Plaque erosion causing ST-elevation myocardial infarction after consumption of cannabis and N_2_O in a 27-year old man: a case report

**DOI:** 10.1186/s12872-021-01953-3

**Published:** 2021-03-23

**Authors:** Sarah Bär, Fabien Praz, Lorenz Räber

**Affiliations:** Department of Cardiology, Bern University Hospital Inselspital, University of Bern, 3010 Bern, Switzerland

**Keywords:** Case report, Optical coherence tomography, ST-elevation myocardial infarction, Percutaneous coronary intervention, Plaque erosion, Drug abuse

## Abstract

**Background:**

The recreational drugs cannabis and nitrous oxide (N_2_O) are known for pro-atherogenic effects and are associated with an elevated risk of myocardial infarction. These cardiovascular effects might be underestimated by the public. Culprit-lesion composition of myocardial infarctions associated with cannabis and N_2_O has been unknown so far. This case report aims to raise the awareness of the adverse cardiovascular effects of cannabis and N_2_O and reports, for the first time, optical coherence tomography (OCT) findings of the culprit lesion.

**Case presentation:**

This is a case report of a 27-year old man with anterior ST-segment-elevation myocardial infarction (STEMI) after intoxication with cannabis and N_2_O. Coronary angiography and OCT revealed plaque erosion with subsequent subtotal thrombotic occlusion of the left anterior descending artery that was successfully treated with 1 drug-eluting stent. The patient was symptom free at 6 months follow-up and had been able to abstain from drug consumption.

**Conclusions:**

This is the first case to demonstrate the association between cannabis and N_2_O abuse and plaque erosion on OCT in a young man with STEMI. In contrast to smoking, whose adverse effects are well-known, the cardiovascular effects of cannabis and N_2_O might be underestimated. These adverse effects should gain more awareness in the public to prevent early vascular events in young adults.

**Supplementary Information:**

The online version contains supplementary material available at 10.1186/s12872-021-01953-3.

## Background

Cannabis and nitrous oxide (N_2_O) [1] are increasingly consumed as recreational drugs. Both substances are known for pro-atherogenic effects [2–4] and are associated with an elevated risk of myocardial infarction [5, 6]. These adverse cardiovascular effects might be underestimated by the public. Furthermore, details of the culprit-lesion composition in these rare myocardial infarctions in the young have been unknown so far.

## Case presentation

**Fig. 1:**
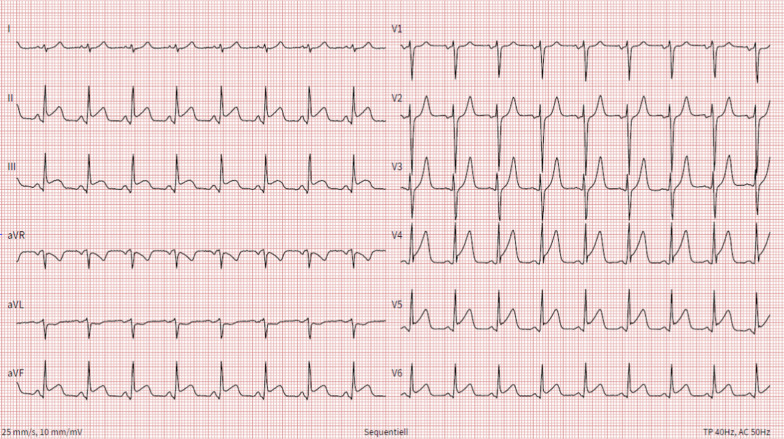
ECG at hospital admission. ECG = electrocardiogram

**Fig. 2:**
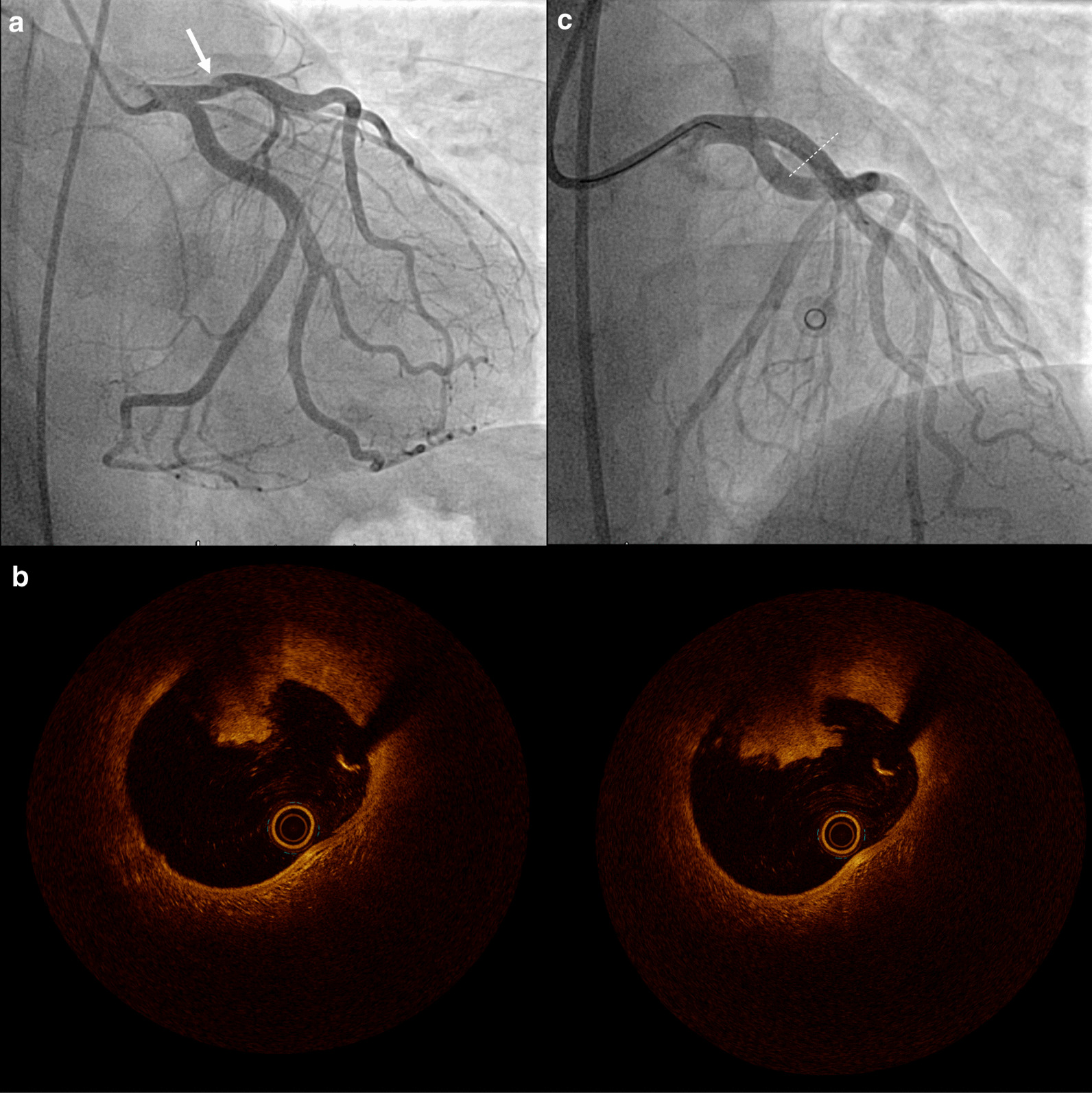
Angiography and OCT findings. **a** Thrombotic lesion in the proximal LAD, **b** adhesive non-occlusive predominantly white thrombi superimposed on a fibrous plaque in 2 consecutive OCT frames, **c** final angiographic result after stenting. LAD = left anterior descending artery, OCT = optical coherence tomography

A 27-year old man presented to the emergency department with acute chest pain for 2 h. Blood pressure was 115/78 mmHg and heart rate 113/min. Heart and lung sounds were clear and radial and pedal pulses symmetrically palpable. Electrocardiogram (ECG) showed ST-elevation in leads II, III, avF, V4-V6 (Fig. 1). The patient admitted consumption of alcohol, cannabis and N_2_O in the hours before symptom onset. His medical history was notable for Hodgkin-Lymphoma treated with curative radio-chemotherapy 3 years previously. Differential diagnosis of ST-elevations and chest pain in a young male include myocardial infarction due to a non-atherosclerotic origin (i.e. coronary embolism, cocaine induced vasospasm), spontaneous coronary artery dissection, peri-myocarditis, early repolarization, or pneumothorax. The patient was referred for urgent coronary angiogram, which revealed a thrombotic lesion in the proximal left anterior descending artery (LAD) (Fig. 2, panel A, white arrow; Additional File 1: Fig. S1; Additional File 2: Video S1). Intracoronary OCT yielded no plaque rupture, but adhesive non-occlusive predominantly white thrombi superimposed on a fibrous plaque, suggestive of plaque erosion [7] (Fig. 2, panel B; Additional File 3: Video S2). Distal embolization of the thrombus material was suspected to be the correlate for the concomitant inferior ST-elevations observed on initial ECG. Left ventricular function was 55% with apical akinesia. Laboratory analysis yielded normal hemoglobin and thrombocyte count, normal INR, an alcohol plasma level of 1.7 per mill and newly diagnosed dyslipidemia (total cholesterol 5.3 mmol/l, low-density lipoprotein cholesterol (LDL-C) 3.9 mmol/l). High-sensitivity troponin T on admission was 180 ng/l and peak level 896 ng/l. Screening for anti-phospholipid syndrome was negative and urine toxicology confirmed the consumption of tetrahydrocannabinol, but was negative for other illicit drugs including cocaine. Transthoracic echocardiogram showed normal biventricular function, normal dimensions of all cardiac cavities, no thrombi, and normal valve function.

The lesion in the LAD was successfully treated with direct implantation of a XIENCE Sierra™ 4.0/18 mm drug-eluting stent and postdilatation with a 4.0/15 mm non-compliant balloon with good result on angiography (Fig. 1, panel C; Additional File 2: Video S1). Dual-antiplatelet therapy with aspirin long-term and the potent P2Y12 inhibitor prasugrel for 1 year, as well as high-intensity lipid lowering therapy, betablocker (IIaB recommendation [8]) and ACE-inhibitor (IIaA recommendation [8]) were initiated, in accordance with current recommendations [8]. The patient underwent lifestyle change counseling with emphasis on smoking cessation and information on the adverse effects of cannabis and N_2_O consumption. The course of hospitalization was uneventful and the patient was discharged to home after 48 h rhythm monitoring.

At 6-months follow-up, the patient reported good recovery after STEMI with no complaints of chest pain or dyspnoe. He had been actively engaged in an outpatient cardiac rehabilitation program 3 times per week and been able to abstain from drug consumption and reduce smoking (Table 1).

**Table 1:** Timeline

4 h	Consumption of cannabis, N_2_O, and alcohol
2 h	Chest pain onset
0 h	Presentation to the emergency department
10 min	ST-elevation in leads II, III, avF, V4-V6 on initial ECG
2 h	Coronary angiogram and OCT confirming the diagnosis of STEMI due to plaque erosion in the proximal LAD
2 h 25 min	Successful implantation of 1 drug-eluting stent into the LAD
1–2 days	Uneventful rhythm monitoring
2 days	Hospital discharge
6 months	Good recovery after STEMI, cessation of drug consumption, reduction of smoking, had participated in outpatient cardiac rehabilitation program

*ECG* electrocardiogram, *LAD* left anterior descending artery, *STEMI* ST-segment elevation myocardial infarction

## Discussion and conclusions

Cannabis is known for pro-atherogenic effects by generation of reactive oxygen species with promotion of endothelial injury [2] and lipid accumulation in macrophages [3] and has been associated with a 4.8-fold increased risk of myocardial infarction within 1 h of its use [6]. N_2_O leads to an elevation of plasma homocysteine levels [4] and acute elevations of homocysteine levels are associated with endothelial dysfunction, oxidative stress, enhanced platelet activation and vascular inflammation [9]. N_2_O-based anesthesia was shown to be associated with an increased risk of postoperative myocardial ischemia [4]. Furthermore, an elevated risk of myocardial infarction even through long-term follow-up of 3.5 years has been observed (adjusted odds ratio 1.59, p = 0.04) [5]. Outside the operating room, where N_2_O is used as a general anesthetic, N_2_O is being increasingly consumed as recreational drug [1]. In 2015, N_2_O was the second most popular recreational drug after cannabis in the United Kingdom. In most countries, N_2_O is legal, cheap, and widely available. N_2_O is mostly inhaled from a balloon and induces an euphoric, empathogenic and sometimes a hallucinogenic state [1].

The association between N_2_O intoxication and STEMI in a young man has been reported previously [10], however, to our knowledge, this is the first case, to report plaque erosion on OCT in a young man with STEMI after consumption of cannabis and N_2_O. This patient has 2 classical cardiovascular risk factors (smoking, dyslipidemia) and the risk for myocardial infarction is known to be elevated in survivors of Hodgkin-Lymphoma compared to age- and sex-matched controls [11]. However, acute toxic effects of cannabis and N_2_O causing myocardial infarction [6] and ischemia [4] have been reported, and the plaque erosion on OCT may imply, that the combined acute vascular effects of both drugs may have triggered endothelial denudation with subsequent thrombus formation and STEMI in this young man. Further research is warranted to investigate this potential causal relationship.

In a recent study on 153 patients with erosion and ACS, local inflammation as expressed by the presence of macrophage infiltration on OCT, was associated with significantly more unstable plaque features and major adverse cardiac events through 2.5 years follow-up. However, only 33.3% of the patients exhibited macrophages on OCT, while 66.7% did not [12]. Consistent with the majority of patients in this study, the patient from the present case did not show macrophage infiltration on OCT. Laboratory analysis yielded normal C-reactive protein, but elevated white blood cell count (15.2 G/l) on admission. The role of local and systemic inflammation in erosion should be investigated in further studies.

Plaque erosion is responsible for 25–40% of acute coronary syndromes and is more frequently observed in younger patients and smokers with a lower prevalence of hypertension, diabetes mellitus and dyslipidemia [7]. A potential paradigm shift towards anti-thrombotic medical management without stent implantation in coronary plaque erosion, is a current matter of debate [7, 13]. A proof-of-concept study showed favorable outcomes with a non-stenting strategy in plaque erosion [13]. This may be of particular importance in young patients like the present case, where the long-term sequela after stent implantation could potentially be prevented. However, data are still too limited to provide general recommendations [8, 14]. Therefore, to adhere to current guideline recommendations, this patient was treated with stent implantation.

OCT is the pivotal diagnostic tool to unravel the underlying pathophysiology in atypical presentations with acute coronary syndromes as the present case. True atherosclerotic plaque rupture can be distinguished from plaque erosion, spontaneous coronary artery dissection, coronary thromboembolism, or coronary spasm. These entities can be difficult/impossible to differentiate on angiography, and OCT may be required to establish the correct diagnosis and initiate appropriate therapy [14].

In contrast to smoking, whose adverse effects are well-known to the public, the cardiovascular effects of recreational drugs like cannabis and N_2_O might be underestimated. In the light of previous reports [4, 5, 10] and the present case, the increasing use of the cheap, widely available, and in most countries legal drug N_2_O [1] is alarming. Thus, the adverse vascular effects of cannabis and especially N_2_O should gain more awareness in the public to prevent early vascular events in young adults.

The strength of this case report is the investigation of the culprit-lesion with OCT, which is unique in the field. The main limitation is that a causal relationship between cannabis and N_2_O consumption and plaque erosion cannot be established from this single report, especially in the presence of other cardiovascular risk factors.

In conclusion, this is the first report of plaque erosion on OCT in a young man with STEMI after consumption of cannabis and N_2_O.

## Supplementary Information

**Additional file 1: Fig. S1.** Angiographic projections. Additional projections of the left coronary system (A–D) and the right coronary artery (E–F).

**Additional file 2: Video S1.** Clips of coronary angiography and percutaenous coronary intervention.

**Additional file 3: Video S2**. OCT pullback. OCT = optical coherence tomography.

## Data Availability

Data sharing is not applicable to this article as no datasets were generated or analysed during the current study.

## Abbreviations

ECG: Electrocardiogram
LAD: Left anterior descending artery
LDL-C: Low-density lipoprotein cholesterol
N_2_O: Nitrous oxide
OCT: Optical coherence tomography
STEMI: ST-segment elevation myocardial infarction

## References

[1] van Amsterdam J, Nabben T, van den Brink W (2015). Recreational nitrous oxide use: prevalence and risks. Regul Toxicol Pharmacol.

[2] Tiyerili V, Zimmer S, Jung S, Wassmann K, Naehle CP, Lütjohann D (2010). CB1 receptor inhibition leads to decreased vascular AT1 receptor expression, inhibition of oxidative stress and improved endothelial function. Basic Res Cardiol.

[3] Jiang L, Pu J, Han Z, Hu L, He B (2009). Role of activated endocannabinoid system in regulation of cellular cholesterol metabolism in macrophages. Cardiovasc Res.

[4] Badner NH, Beattie WS, Freeman D, Spence JD (2000). Nitrous oxide-induced increased homocysteine concentrations are associated with increased postoperative myocardial ischemia in patients undergoing carotid endarterectomy. Anesth Analg.

[5] Leslie K, Myles PS, Chan MTV, Forbes A, Paech MJ, Peyton P (2011). Nitrous oxide and long-term morbidity and mortality in the ENIGMA trial. Anesth Analg.

[6] Mittleman MA, Lewis RA, Maclure M, Sherwood JB, Muller JE (2001). Triggering myocardial infarction by marijuana. Circulation.

[7] Partida RA, Libby P, Crea F, Jang I-K (2018). Plaque erosion: a new in vivo diagnosis and a potential major shift in the management of patients with acute coronary syndromes. Eur Heart J.

[8] Ibanez B, James S, Agewall S, Antunes MJ, Bucciarelli-Ducci C, Bueno H (2018). 2017 ESC Guidelines for the management of acute myocardial infarction in patients presenting with ST-segment elevation. Eur Heart J.

[9] Pushpakumar S, Kundu S, Sen U (2014). Endothelial dysfunction: the link between homocysteine and hydrogen sulfide. Curr Med Chem.

[10] Indraratna P, Alexopoulos C, Celermajer D, Alford K (2017). Acute ST-elevation myocardial infarction, a unique complication of recreational nitrous oxide use. Heart Lung Circ.

[11] Bhakta N, Liu Q, Yeo F, Baassiri M, Ehrhardt MJ, Srivastava DK (2016). Cumulative burden of cardiovascular morbidity in paediatric, adolescent, and young adult survivors of Hodgkin’s lymphoma: an analysis from the St Jude Lifetime Cohort Study. Lancet Oncol.

[12] Montone RA, Vetrugno V, Camilli M, Russo M, Fracassi F, Khan SQ (2020). Macrophage infiltrates in coronary plaque erosion and cardiovascular outcome in patients with acute coronary syndrome. Atherosclerosis.

[13] Xing L, Yamamoto E, Sugiyama T, Jia H, Ma L, Hu S (2017). EROSION Study (effective anti-thrombotic therapy without stenting: intravascular optical coherence tomography-based management in plaque erosion): a 1-year follow-up report. Circ Cardiovasc Interv.

[14] Johnson TW, Räber L, de Mario C, Bourantas C, Jia H, Mattesini A (2019). Clinical use of intracoronary imaging. Part 2: acute coronary syndromes, ambiguous coronary angiography findings, and guiding interventional decision-making: an expert consensus document of the European Association of Percutaneous Cardiovascular Interventions. Eur Heart J.

